# Pharmacological treatment of postoperative recurrence of Crohn’s disease: Protocol for systematic review and network meta-analysis

**DOI:** 10.1371/journal.pone.0310752

**Published:** 2024-10-09

**Authors:** Tianxiang Jiang, Zhaolun Cai, Chunjuan Liu, Bo Zhang

**Affiliations:** 1 Department of General Surgery, West China Hospital, Sichuan University, Chengdu, China; 2 Gastric Cancer Center, West China Hospital, Sichuan University, Chengdu, China; University of Hertfordshire, UNITED KINGDOM OF GREAT BRITAIN AND NORTHERN IRELAND

## Abstract

**Background:**

Crohn’s disease (CD) is a chronic inflammatory condition primarily affecting the digestive system. When dealing with complex cases like intestinal blockages or perforations, surgery becomes the primary treatment option. However, surgery doesn’t offer a complete cure, and the possibility of recurrence remains. To manage CD recurrence after surgery, various treatment choices are available, including steroids, monoclonal antibodies, immunomodulators, and further surgery. Regrettably, the current body of evidence doesn’t definitively establish which of these treatments is the most effective and safe. Thus, our research aims to provide insights into the Validity and security of different treatment approaches for managing CD recurrence after surgery.

**Methods:**

Search of EMBASE, PubMed, Web of Science Core Collection and the Cochrane Central Register of Controlled Trials will be conducted to include researches that examine the validity of treatments for recurrent CD after surgery. Our analysis will distinguish between two types of studies: randomized controlled trials (RCTs) and non-randomized studies with at least two different treatments, each evaluated separately. We will employ Bayesian network meta-analyses to systematically compare the effectiveness and safety of these treatments. Additionally, subgroup analyses will be performed according to recurrence status and postoperative prophylactic medication. To clarify the variation of studies, sensitivity analyses will be performed. And we may use meta-regression as an additional approach if relevant data are available. We will also rigorously access the certainty of evidence using the Grading of Recommendations, Assessment, Development, and Evaluation framework.

**Discussion:**

This analysis will provide a comprehensive assessment of the latest evidence on available treatments for patients with postoperative recurrence of CD, which will provide recommendations for clinical practice.

**Trial registration:**

**Systematic review registration**

INPLASY2023110021. (DOI: 10.37766/inplasy2023.11.0021).

## Background

Crohn’s disease (CD) is a chronic inflammatory condition primarily affecting the gastrointestinal tract, with the most common sites of manifestation being the terminal ileum and colon [1]. The persistent and recurrent nature of CD not only affects patients’ overall quality of life but also has the potential to impact their psychological well-being [2]. Management of CD is multifaceted, with treatment choices depending on the disease’s severity and the patient’s risk assessment. Treatment options include a range of approaches, such as steroid therapy, monoclonal antibody therapy, immunomodulators, and surgical procedures [3,4]. With the evolving landscape of treatment options, emerging interventions like certolizumab pegol [5], ustekinumab [6], Risankizumab [7], and stem cell transplantation [8] have gained attention and initial assessments. For patients dealing with complex conditions like bowel stenosis and/or perforation, surgical intervention is often the preferred therapeutic option [9]. However, it’s important to emphasize that surgery doesn’t guarantee a complete cure for CD. 48% of CD patients experienced clinical recurrence after surgery (median follow-up period: 88 months) [10].

Postoperative recurrences in CD can manifest in two forms: either endoscopic or clinical recurrences, with endoscopic recurrence typically preceding clinical symptoms [11]. Therefore, it is crucial to incorporate routine, periodic endoscopic evaluations into the postoperative management of CD patients [12]. Numerous studies have identified various risk factors associated with postoperative CD recurrence. These risk factors include involvement of the ileocolonic region, the presence of penetrating disease, prior perianal disease, a history of smoking, positive surgical margins, the presence of granulomas, and mesenteric plexitis [13–17]. Stratifying patients based on these recognized risk factors holds promise for tailoring personalized preventive management strategies, ultimately improving patient outcomes [18]. A comprehensive pooled analysis has demonstrated the superior effectiveness of anti-tumor necrosis factor (TNF)-α therapy in comparison to azathioprine prophylaxis for preventing postoperative CD recurrence [19]. In line with this, a network meta-analysis published in 2019 reaffirms the efficacy of anti-TNF-α therapy, either as a standalone treatment or combination with other approaches, as the most promising therapeutic option for preventing recurrence following endoscopic surgery in CD patients [20]. The surgical approach also has an impact on the recurrence rate. It was found that the Kono-S anastomosis might reduce the postoperative endoscopic recurrence rate and the clinical recurrence rate compared with the standard anastomosis method [21].

Numerous studies have explored various treatment options to address postoperative recurrence in CD. Notably, two Randomized Controlled Trials (RCTs) found no significant difference in treatment failure rates between azathioprine and mesalazine for postoperative endoscopic recurrence in CD [22,23]. However, it’s important to emphasize that azathioprine is associated with a less favorable safety profile [22]. In a prospective study designed to compare the therapeutic effectiveness of infliximab, mesalazine, and azathioprine for postoperative endoscopic recurrence, the results showed that infliximab treatment had a significant inhibitory effect on both clinical and endoscopic disease activity in patients who had undergone resection and had early-stage endoscopic lesions [24]. Additionally, patients treated with infliximab displayed significantly higher rates of endoscopic response and remission compared to those receiving adalimumab [25]. Despite these valuable insights, it’s crucial to acknowledge that comprehensive and definitive guidance on the treatment of postoperative CD recurrence remains elusive.

Hence, this network meta-analyses and systematic reviews aims to comprehensively amalgamating all available evidence. This approach not only expands the sample size but also streamlines the process of identifying the most effective strategies among the treatments mentioned. Network meta-analysis differs from traditional meta-analysis with the unique advantage of covering a wider range of published studies. This is achieved by enabling not only direct comparisons but also indirect comparisons among three or more methods. In order to provide more guidance for clinical decision-making, certainty of evidence is going to be assessed through the Grading of Recommendations, Assessments, Developments and Evaluations (GRADE) tool [26].

## Methods

### Design

The guidelines outlined in the Preferred Reporting Items for Systematic Reviews and Meta-Analyses Protocols (PRISMA-P) were followed by the study [27]. The forthcoming formal study is following the guidance provided by the Preferred Reporting Items for Systematic Reviews and Meta-Analyses-Network Meta-Analysis (PRISMA-NMA) checklist [28].

### Information resources and search strategy

A comprehensive search of EMBASE, PubMed, Web of Science Core Collection and the Cochrane Central Register of Controlled Trials will be conducted. The timeframe is from their inception to January 2024.There will be no language restrictions. To ensure thorough coverage, the search will incorporate a combination of Medical Subject Headings (MeSH) terms, text words, and their synonyms. Additionally, we will perform manual searches and examine references to expand the search scope. Supplementary 1 lists the draft search strategy for PubMed.

### Eligibility/Exclusion criteria

**Participants.** Patients meeting the following criteria will be included:

- Confirmed diagnosis of CD through pathology;
- Underwent ileocolonic resection for CD;
- Experience postoperative recurrence, either endoscopic or clinical.

#### Interventions

Researches focusing on comparing at least two of the following different pharmacological treatments are eligible: corticosteroids, sulfasalazine, mesalazine, azathioprine, methotrexate, vedolizumab, infliximab, adalimumab, certolizumab pegol, ustekinumab, risankizumab, upadacitinib, and antibiotics.

#### Outcomes

The primary outcomes include:

- Endoscopic remission. A decrease of at least one point in the Rutgeerts score is considered endoscopic remission.
- For endoscopic recurrence, the outcome is the clinical recurrence rate.
- For clinical recurrence, achieving clinical remission (based on the CD Activity Index (CDAI) or Harvey-Bradshaw Index) and serological remission (including CRP, ESR, FC, etc.).

The secondary outcomes consist of:

- Treatment failure, for various reasons such as adverse events, disease recurrence, reoperation, or others.
- Incidence of adverse events.

The time points for the above outcome assessments were 6, 12, 18 and 24 months.

#### Study designs

This study will include both non-randomized studies (NRSs) and randomized controlled trials (RCTs), with NRSs including prospective or retrospective cohort studies. Both of them will be analyzed separately. We will include full-text publications, trials with no commercial purpose, and abstracts with sufficient information.

Exclusion criteria:

- Excluded studies are reviews, letters, commentaries, conference abstracts and studies on animals.
- Studies without predefined outcomes will be excluded.
- Studies with duplicated patients from the same organization will be excluded unless we can verify that individual patients do not overlap by checking the inclusion criteria and study duration. If overlap is confirmed, the author will be contact for clarification. Otherwise, we will include only the most current or comprehensive content.

### Study selection

Titles and abstracts of all research are going to be viewed by two authors (TJ and CL) for eligibility, independently. If any articles raise questions about eligibility, they will be carefully reviewed in full text to gather additional information. Any conflicts would be settled in consultation with the other author, BZ.

### Data extraction

The following information will be extracted from the selected study using a standardized form: first author, publication year, sample size, study design, follow-up time, treatment regimen, endoscopic response and endoscopic remission (as indicated by changes in Rutgeerts’ score) and the number and rate of clinical recurrences, clinical remissions, serological remissions, treatment failures, adverse events. Preoperative treatments received (anti-TNF; one, two or more biologics) and postoperative prophylactic medications (yes or no, and specific medication regimen) will also be recorded in detail. Any instances involving the settlement of disputes through negotiation or communication with the original author will be recorded in detail. In addition, reasons for exclusion of any study after view the full article will also be documented to improve reproducibility, and would be placed in the supplementary material.

### Risk of bias assessment

#### For RCTs

For randomized controlled trials, the Cochrane risk-of-bias tool 2.0 [29,30] will be employed. Six dimensions of bias will be assessed: randomization, blinding, allocation concealment, selective reporting, incomplete reporting of results and other. Ultimately, every research will be classified as high, low risk of bias, or unclear risk of bias.

#### For NRSs

To assess the risk of bias in NRSs, we will employ the Risk of Bias in Non-randomized Studies of Interventions (ROBINS-I) tool [31]. It thoroughly evaluates seven areas of bias encompassing the entire intervention process, including (1) confounders; (2) participant selection; (3) intervention categorization; (4) deviations from the intended intervention; (5) missing data; (6) outcome measures; and (7) selective reporting of results. Each study will be assessed for bias and considered for classification into four levels of risk of bias. Studies missing key information will be considered to have no information.

For risk of bias in observational epidemiological studies, the Risk Of Bias In Non-randomized Studies of Exposure (ROBINS-E) tool [32] will be used. It thoroughly evaluates seven areas of bias encompassing the entire intervention process, including (1) confounding; (2) measurement of the exposure; (3) participants; (4) post-exposure interventions; (5) missing data; (6) outcome measures; and (7) selective reporting of results. Each study will be assessed for bias and considered for classification into four levels of risk of bias. Studies missing key information will be considered to have no information.

Bias evaluation is going to be done independently by two authors (TJ and CL). Any conflicts will be settled by negotiation with another author, BZ, until reaching a consensus.

### Assessment of small sample effects

The impact of small sample sizes on the results will be assessed by plotting the comparison-adjusted funnel plots [33].

### Processing of missing data

In case of missing data, efforts will be made to seek complete data from the corresponding author. If no response, the Cochrane Handbook for Systematic Reviews will be referred to for estimating missing statistics using availability coefficients. For continuity results, the standard deviation (SD) will be estimated using available information such as the standard error, p-value, or confidence interval, depending on what was provided in the original study. Else, SD was estimated according to the median or interquartile range (IQR) [34]. Sensitivity analyses are going to be conducted to evaluate the potential impact of these missing data.

### Statistical analyses

In cases where quantitative analyses were not feasible, we provided a descriptive narrative of the results. If quantitative analyses are viable, we will proceed with subsequent statistical analyses. The randomized controlled trials (RCTs) and non-randomized studies (NRSs) will be synthesized and analyzed separately [35].

#### Network geometry

We will create network plots to visually depict the various intervention types, which include corticosteroids, sulfasalazine, mesalazine, azathioprine, methotrexate, vedolizumab, infliximab, adalimumab, certolizumab pegol, ustekinumab, risankizumab, upadacitinib, and antibiotics.

#### Transitivity assessment

Narrative summaries will be provided for each included study. The distribution of baseline characteristics of participants in different studies and treatments will be compared to ensure comparability. Considerable heterogeneity will be indicated by I^2^ > 50% or a p < 0.1.

#### Direct comparisons

Traditional paired meta-analyses will be conducted with STATA V.12.0 software if an outcome is available in at least two studies. Random effects models and the DerSimonian-Laird method will be applied for analysis [36]. The χ^2^ test and I^2^ statistic will quantify the level of inter-trial heterogeneity.

#### Mixed & indirect comparison

The Bayesian Markov Chain Monte Carlo framework and the gemtc package of R software would be employed for the network meta-analysis [37,38]. Odds ratios (OR) and 95% credible intervals (CrI) will be used to evaluate dichotomous data. Analysis of continuous variables will be performed according to the scale of measurement, using either weighted mean differences or standardized mean differences. The surface under the cumulative sorted area value will be employed to sort the different interventions [39,40].

#### Inconsistency evaluation

Inconsistency assessments will be used to identify differences between direct and indirect comparisons in analyses involving both. Inconsistency will be evaluated through node splitting, where mixed evidence of nodes is divided into direct and indirect evidence for comparison [41,42]. If no discrepancies are found, the consistency model is applicable. In contrast, if significant differences exist, we will employ inconsistency modeling and further discuss potential reasons for inconsistency.

#### Subgroup analysis

Subgroup analyses will be employed to interpret the sources of heterogeneity. If data are available, we may also use meta-regression as an additional method. Preliminary subgroupings include recurrence status (endoscopic or clinical), preoperative treatment (anti-TNF; one, two, or more biologics), and postoperative prophylactic medications (yes or no, and specific medication regimen).

#### Sensitivity analysis

Sensitivity analyses will be conducted, if available, to assess the stability of the results through removal of studies at high risk of bias.

### Certainty of evidence

The GRADE tool will be used to assess certainty of the evidence, which will be employed to assign quality ratings to treatment effect estimates [26]. Very low, low, moderate or high levels of certainty of evidence will be assigned, taking into account factors such as risk of bias, publication bias, imprecision, inconsistency, and indirectness.

## Discussion

This systematic review and network meta-analysis aims to provide a comprehensive assessment of the latest evidence on the optimal pharmacotherapy of postoperative CD recurrence patients. Our analysis will consider the efficacy (Rutgeerts’ score, clinical recurrence rate for endoscopic recurrence, and clinical remission rate for clinical recurrence) and safety (treatment failures and adverse events) of available treatments. By summarizing and analyzing the evidence, our study will not only provide guidance for treatment decisions in clinical practice, but also provide direction for future research.

## Supporting information

S1 File Search strategy for PubMed. (DOCX)

S2 File PRISMA checklist. (DOC)

S3 File PRISMA flow diagram. (DOCX)
