# Supplementary material for: Pharmacological treatment of postoperative recurrence of Crohn’s disease: Protocol for systematic review and network meta-analysis
Source: PLoS One. 2024 Oct 9;19(10):e0310752. doi: 10.1371/journal.pone.0310752 (PMC11463762; doi:10.1371/journal.pone.0310752)
Supplement: S1 File — (DOCX) [file pone.0310752.s001.docx]

**Treatment of Postoperative Recurrence of Crohn’s Disease: A Protocol of Systematic Review and Network Meta-analysis**

Running Title: Treatment of Postoperative Recurrence of Crohn’s Disease

Tianxiang Jiang, Zhaolun Cai, Chunjuan Liu, Bo Zhang

**Supplementary Materials 1: Search strategy for PubMed.**

1. "crohn disease"[MeSH Terms] OR ("crohn"[All Fields] AND "disease"[All Fields]) OR "crohn disease"[All Fields] OR "crohn s disease"[All Fields]
2. "postoperative period"[MeSH Terms] OR ("postoperative"[All Fields] AND "period"[All Fields]) OR "postoperative period"[All Fields] OR "postop"[All Fields] OR "postoperative"[All Fields] OR "postoperatively"[All Fields] OR "postoperatives"[All Fields]
3. "recurrance"[All Fields] OR "recurrence"[MeSH Terms] OR "recurrence"[All Fields] OR "recurrences"[All Fields] OR "recurrencies"[All Fields] OR "recurrency"[All Fields] OR "recurrent"[All Fields] OR "recurrently"[All Fields] OR "recurrents"[All Fields]
4. "adrenal cortex hormones"[MeSH Terms] OR ("adrenal"[All Fields] AND "cortex"[All Fields] AND "hormones"[All Fields]) OR "adrenal cortex hormones"[All Fields] OR "corticosteroid"[All Fields] OR "corticosteroids"[All Fields] OR "corticosteroidal"[All Fields] OR "corticosteroide"[All Fields] OR "corticosteroides"[All Fields]
5. "sulfapyridine"[MeSH Terms] OR "sulfapyridine"[All Fields] OR "sulphapyridine"[All Fields]
6. "azathioprin"[All Fields] OR "azathioprine"[MeSH Terms] OR "azathioprine"[All Fields]
7. "methotrexate"[MeSH Terms] OR "methotrexate"[All Fields] OR "methotrexate's"[All Fields] OR "methotrexates"[All Fields]
8. "vedolizumab"[Supplementary Concept] OR "vedolizumab"[All Fields]
9. "infliximab"[MeSH Terms] OR "infliximab"[All Fields] OR "infliximab's"[All Fields]
10. "adalimumab"[MeSH Terms] OR "adalimumab"[All Fields]
11. "certolizumab pegol"[MeSH Terms] OR ("certolizumab"[All Fields] AND "pegol"[All Fields]) OR "certolizumab pegol"[All Fields] OR "certolizumab"[All Fields]
12. "ustekinumab"[MeSH Terms] OR "ustekinumab"[All Fields]
13. "risankizumab"[Supplementary Concept] OR "risankizumab"[All Fields]
14. "upadacitinib"[Supplementary Concept] OR "upadacitinib"[All Fields]
15. "anti bacterial agents"[Pharmacological Action] OR "anti bacterial agents"[MeSH Terms] OR ("anti bacterial"[All Fields] AND "agents"[All Fields]) OR "anti bacterial agents"[All Fields] OR "antibiotic"[All Fields] OR "antibiotics"[All Fields] OR "antibiotic s"[All Fields] OR "antibiotical"[All Fields]
16. #4 OR #5 OR #6 OR #7 OR #8 OR #9 OR #10 OR #11 OR #12 OR #13 OR #14 OR #15
17. #1 AND #2 AND #3 AND #16
